# Plasma diacylglycerol composition is a biomarker of metabolic syndrome onset in rhesus monkeys[S]

**DOI:** 10.1194/jlr.M057562

**Published:** 2015-08

**Authors:** Michael A. Polewski, Maggie S. Burhans, Minghui Zhao, Ricki J. Colman, Dhanansayan Shanmuganayagam, Mary J. Lindstrom, James M. Ntambi, Rozalyn M. Anderson

**Affiliations:** * Department of Medicine, University of Wisconsin, Madison, WI; † Department of Biochemistry, University of Wisconsin, Madison, WI; § National Primate Research Center, University of Wisconsin, Madison, WI; ** Department of Biostatistics and Medical Informatics, University of Wisconsin, Madison, WI; †† Department of Nutritional Sciences, University of Wisconsin, Madison, WI; §§ Geriatric Research, Education, and Clinical Center, William S. Middleton Memorial Veterans Hospital, Madison WI

**Keywords:** lipids, lipoproteins, insulin, obesity, adipose tissue, cholesteryl esters, free fatty acids, phospholipids, triacylglycerols, adiponectin

## Abstract

Metabolic syndrome is linked with obesity and is often first identified clinically by elevated BMI and elevated levels of fasting blood glucose that are generally secondary to insulin resistance. Using the highly translatable rhesus monkey (*Macaca mulatta*) model, we asked if metabolic syndrome risk could be identified earlier. The study involved 16 overweight but healthy, euglycemic monkeys, one-half of which spontaneously developed metabolic syndrome over the course of 2 years while the other half remained healthy. We conducted a series of biometric and plasma measures focusing on adiposity, lipid metabolism, and adipose tissue-derived hormones, which led to a diagnosis of metabolic syndrome in the insulin-resistant animals. Plasma fatty acid composition was determined by gas chromatography for cholesteryl ester, FFA, diacylglycerol (DAG), phospholipid, and triacylglycerol lipid classes; plasma lipoprotein profiles were generated by NMR; and circulating levels of adipose-derived signaling peptides were determined by ELISA. We identified biomarker models including a DAG model, two lipoprotein models, and a multiterm model that includes the adipose-derived peptide adiponectin. Correlations among circulating lipids and lipoproteins revealed shifts in lipid metabolism during disease development. We propose that lipid profiling may be valuable for early metabolic syndrome detection in a clinical setting.

Metabolic dysfunction is associated with increased vulnerability for a spectrum of diseases (1) and negatively impacts quality of life (2). Although obesity and glucoregulatory dysfunction dominate health care in the United States today, current assessments to identify risk for diabetes rely largely on BMI, waist circumference, and family history. According to the 2010 Centers for Disease Control report, 69% of the adult population is overweight or obese, and 85% of type 2 diabetics are overweight or obese; however, only 10% of the overweight and obese are diabetic. This indicates that glucoregulatory dysfunction is exacerbated by obesity but suggests that the etiology may be more nuanced. One possibility is related to the endocrine nature of adipose tissue, where production of bioactive peptides and fatty acids rather than the expansion of adipose tissue in itself plays a role in disease vulnerability (3). We do not currently have a biochemical marker to accurately predict which overweight/obese individuals are most likely to develop glucoregulatory impairment associated with metabolic syndrome.

The nonhuman primate rhesus macaque (*Macaca mulatta*) is uniquely suited for providing insights into the biology of human diseases. Rhesus monkeys share marked anatomical, physiological, and behavioral similarities with humans, and many diseases and disorders exhibited in humans are also observed in rhesus monkeys (4–7). In addition, conditions that increase in prevalence with advancing age in humans are also manifest in aging rhesus monkeys including neoplasia, sarcopenia, bone loss, loss of immune function, and diabetes (7–9). Importantly, cross-sectional studies confirm that lipoprotein profiles and plasma triacylglycerol (TAG) levels track with metabolic syndrome and diabetes in rhesus monkeys in the same manner as for human clinical evaluations (10, 11).

The goal of this study was to determine early events in the progression to metabolic disease with a focus on lipid metabolism and adipose tissue function. The study cohort included euglycemic overweight rhesus monkeys with insulin resistance in the absence of impaired fasting glucose (IFG) and age and weight pair-matched insulin sensitive controls. Biometric data were analyzed along with plasma adipokine, lipoprotein, and lipid profiles from animals prior to and at the time of spontaneous development of insulin resistance. Differences in measured parameters were evaluated using univariate analysis. The relationship between measured variables and insulin resistance was evaluated using lasso logistic regression to identify statistical models that could accurately distinguish insulin-resistant animals. Models identified using this approach have potential as early biomarkers for disease onset.

## MATERIALS AND METHODS

### Animal care and assessments

Animals were maintained in accordance to guidelines for the ethical care and treatment of animals as approved by the Institutional Animal Care and Use Committee of the Graduate School of the University of Wisconsin-Madison. This study involved 16 adult male rhesus monkeys of Indian origin from 10 to 22 years of age. Animals were housed individually at the Wisconsin National Primate Research Center and were allowed ad libitum access to food for 6–8 h per day. All animals were fed a pelleted, semipurified diet (Teklad, Madison, WI), which contained 15% lactalbumin, 10% corn oil, and ∼65% carbohydrate in the form of sucrose and cornstarch as previously described (12). Animals had continuous access to water and rooms were maintained at 21°C–26°C with ∼50–65% relative humidity. Animals were monitored daily, body weight was monitored weekly, and body composition was monitored every 6 months by dual energy X-ray absorptiometry. Glucoregulatory function was monitored every 6 months using established criteria, where levels of fasting plasma glucose and insulin were determined and insulin sensitivity measured using a frequently sampled intravenous glucose tolerance test (FSIGT) as previously described (6, 13). Plasma samples drawn >3 h following glucose infusion during the FSIGT, a time point when baseline measures of insulin and glucose are reestablished, were stored at −80°C for subsequent analysis as outlined subsequently.

### Lipoprotein profiling

Lipoprotein particles of different sizes were detected in rhesus monkey plasma specimens by NMR spectroscopy using the LipoProfile® test through contract with LipoScience Inc. Analysis also included chemical detection of total HDL, total LDL, total cholesterol, total TAG, and C-reactive protein (CRP), as well as calculated levels for HDL cholesterol and combined total VLDL and chylomicrons.

### Fatty acid composition analysis

Total lipids were extracted from ∼200 μl of fasting plasma according to the method of Bligh and Dyer (14) and separated by silica gel thin layer chromatography using petroleum ether-diethyl ether-acetic acid (80:30:1) as the developing solvent. Lipids derived from trigylcerides, phospholipids (PLs), FFAs, and diacylglycerol (DAG) were scraped, methylated, and analyzed by gas-liquid chromatography on a capillary column coated with DB-225 (30 m length, 0.25 mm, internal diameter, 0.25 µm; Agilent Technologies Inc., Wilmington, DE). Fatty acids were identified by comparison of retention times with authentic standards (Sigma). Pentadecanoic acid (C15:0) was included as an internal standard to control for transmethylation efficiency. Heptadecanoic acid (C17:0) internal standards for each of the analyzed lipid classes were added to samples to allow for calculation of absolute fatty acid concentrations. All fatty acid composition analyses of cholesteryl ester (CE), DAG, FFA, PL, and TAG lipid classes were conducted on n = 8 control and n = 8 impaired for each time point, total 32 specimens. However, because the 17:0 internal standard was omitted from four DAG and FFA samples, these two lipid classes have n = 5 control and n = 7 impaired for the actual concentrations but n = 8 for composition, while CE, PL, and TAG have n = 8 for both composition and concentration.

### Adipokine analysis

For all ELISAs, plasma dilution was optimized for each assay and coefficient of variance of <0.05 was confirmed for replicate measures. Levels of adiponectin and high molecular weight (HMW) adiponectin in plasma were measured using a customization of a commercially available ELISA (DY1065; R and D Systems). Briefly, parallel plasma specimens were incubated in the absence or presence of 0.27 mg/ml proteinase K (Sigma) for 20 min at 37°C to remove lower molecular weight isoforms. Selective digest was confirmed by Western blot. Nondigested isoforms were detected according to the manufacturer’s instructions (detection range 0–4 ng/ml). Divergence from the kit includes use of an alternate capture antibody specific to intact rhesus adiponectin (digested fragments not detected) (AF1065; R and D Systems). Resistin was detected using ELISA (DY1359; R and D Systems) according to the manufacturer’s instructions (detection range 0–2 ng/ml). Leptin was detected using ELISA (MBS705354; MyBioSource) according to the manufacturer’s instructions (detection range 0–2 ng/ml). Specificity of antibodies for rhesus peptides was confirmed by Western blot.

### Statistical approach

Measured variables used in the analysis included biometric measures, fatty acid composition, fatty acid concentration, lipoprotein profiles, and adipokine profiles. The relationships among the measured variables were summarized using rank-based (Spearman) correlations. The correlations that were, in absolute value, ≥0.7, are reported. The association between each response variable and health status, as defined by insulin sensitivity status at time of diagnosis, was evaluated using logistic regression. We report odds ratios (ORs) that summarize the change in odds of impairment for a 1 SD change in the variable. The ability of the groups of measured variables to jointly predict impairment status was assessed using lasso logistic regression fit with cross validation. Lasso regression relates a large number of predictors to a response variable by standard logistic regression subject to the sum of absolute value of the coefficients being smaller than a constant chosen via cross validation. In contrast to the all-or-nothing inclusion of variables used in stepwise regression, lasso allows groups of correlated variables in the model by reducing the impact of each. This produces a more meaningful summary and improved prediction. All analyses were conducted cross-sectionally and longitudinally. Variables measured at 2 years before diagnosis, at time of diagnosis, and the difference calculated for each individual animal between the two time points, each analyzed separately. The R statistical analysis package was used for all analyses and specifically the package glmnet for lasso regression (15).

## RESULTS

### Disease progression is independent of changes in adiposity

**Fig. 1.**
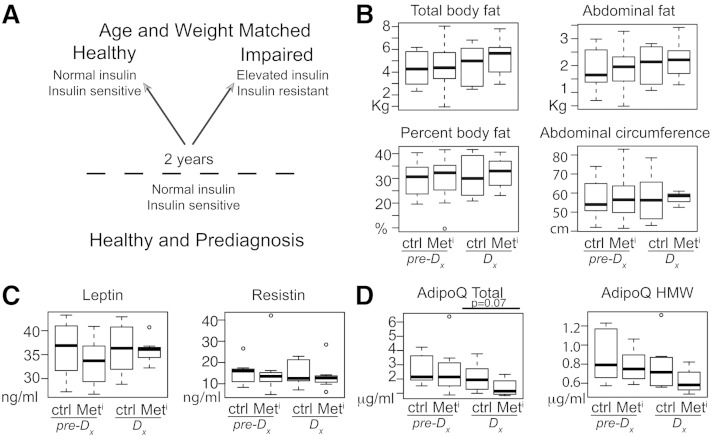
Spontaneous insulin resistance development is independent of a change in adiposity. Schematic of study design, eight animals per group per time point (A); total body fat, percent body fat, abdominal fat, and abdominal circumference (B); plasma leptin and resistin levels (C); and plasma total adiponectin and HMW adiponectin (D) in metabolic impaired (Met^i^) and age- and weight-matched controls (Ctrl) at time of diagnosis (Dx) and 2 years prior to diagnosis (pre-Dx); n = 8 per group per time point. Data are shown as medians and interquartile range (IQR); outliers are shown as open circles. Statistical significance based on univariate analysis (two-sample Wilcoxon).

This study involved a cohort of 16 overweight rhesus monkeys in the age range 10–22 years. Eight animals were identified as insulin resistant when the following criteria were satisfied: fasting insulin >70 μU/ml and insulin sensitivity index (Si) <2(E−04) as determined by FSIGT in combination with an irregular glucose response curve (**Table 1**). The eight control animals were pair matched to impaired animals for age and weight at time of diagnosis but differed in that they had normal levels of fasting insulin and Si >2 with normal glucose response curves. To investigate the trajectory of onset of insulin resistance, we analyzed data and plasma samples from both groups of animals taken at time of diagnosis of insulin-resistant animals and from the same animals 2 years prior to diagnosis when all 16 animals were healthy as defined by the previously discussed metrics (**Fig. 1A**).

**TABLE 1.** Study cohort

	Age (years)	Weight (kg)	Glucose (mg/dl)	Insulin (μU/ml)	Si (E−04)*^a^*
At time of diagnosis					
Healthy	16.82 ± 1.6	14.41 ± 1.6	62.63 ± 2.57	**42.38 ± 7.55**	**3.46 ± 1.09**
Impaired	17.68 ± 1.74	14.76 ± 1.18	85.06 ± 13.45	**146.41 ± 33.27**	**0.54 ± 0.12**
Two years prior to diagnosis					
Healthy	14.83 ± 1.6	13.77 ± 0.86	59.63 ± 2.01	32.88 ± 1.09	3.61 ± 1.09
Preimpaired	15.68 ± 1.73	13.96 ± 1.01	65.16 ± 3.09	48.78 ± 6.72	2.53 ± 0.89

Biometric data means ± SEM (n = 8 per group); values in bold are significantly different (*P* < 0.05).

a Si (E−04) was generated by the modified minimal model approach utilizing data from the FSIGT.

Although the healthy controls were matched by weight to insulin-resistant animals at the time of diagnosis, body composition including abdominal circumference and percent body fat were not part of the selection criteria. Median BMI was not significantly different between healthy and insulin-resistant animals at time of diagnosis or at 2 years prior (supplementary Table 1). Dual energy X-ray absorptiometry measures of body composition revealed no significant differences in total body fat, percent fat, abdominal fat, or percent abdominal fat (not shown) between healthy (Ctrl) and insulin-resistant animals (metabolic impaired [Met^i^]) at time of diagnosis (Dx), or at 2 years prior (pre-Dx) (Fig. 1B). Abdominal circumference (central adiposity) was also not significantly different between groups at either time point. Each of these measures increased with age for both groups, but the difference was not significant. These data show that adiposity alone does not account for the development of insulin resistance.

Next we investigated the possibility that adipose tissue is functionally different in impaired animals compared with healthy controls despite equivalence in adiposity. Adipokines are adipose tissue-secreted peptide signaling molecules that act at the interface of metabolism, inflammation, and immune responses (1, 16, 17). Leptin is a multifunctional protein that plays a role in energy balance, glucose homeostasis, and immunity; its levels correlate positively with adipose tissue mass (18, 19). Resistin is also a proinflammatory molecule with hyperglycemic action (19). Resistin levels are increased in models of obesity, and this has been implicated in the pathogenesis of obesity-associated insulin resistance (16, 18). Circulating levels of leptin and resistin were measured in plasma from metabolically impaired and healthy animals by ELISA, and neither was significantly different between groups at either time point (Fig. 1C). Adiponectin is another adipose-derived signaling hormone that has been associated with metabolic dysfunction and with type 2 diabetes in human studies (20–22). Adiponectin levels have been shown to correlate negatively with adiposity (23); however, in this study adiponectin levels tended to be lower in the impaired animals at time of diagnosis (*P* = 0.07) despite the fact that adiposity and abdominal circumference were equivalent in healthy and insulin-resistant animals. Levels of the HMW isoform of adiponectin also tended to be lower (*P* = 0.10) in impaired animals but were not significantly different between groups at either time point (Fig. 1D).

### Plasma lipoprotein profiles reflect insulin sensitivity status

**Fig. 2.**
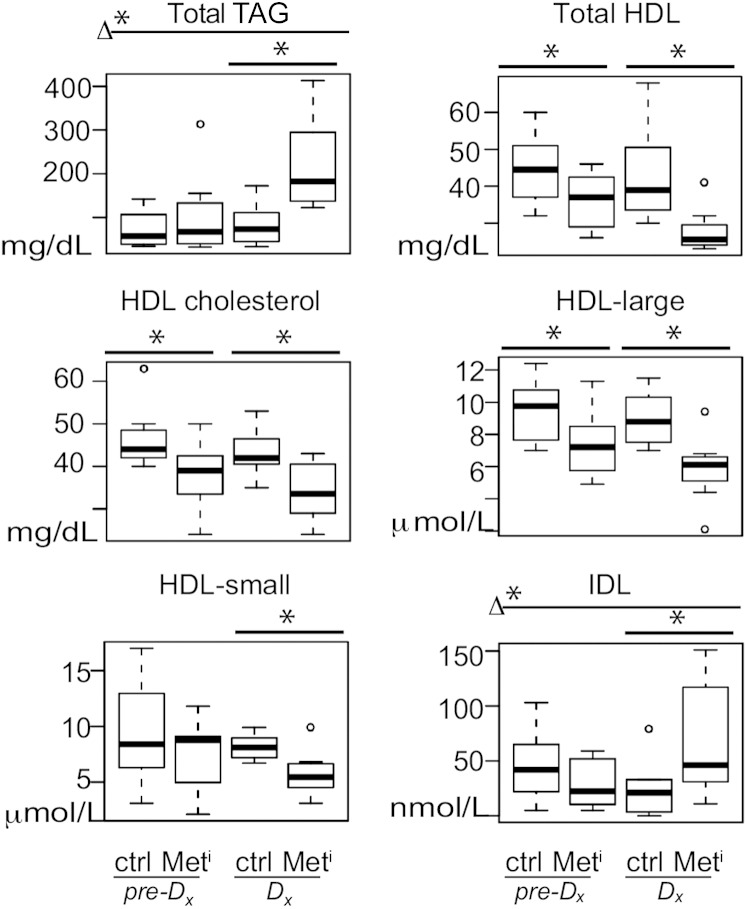
Lipoprotein profiles are altered prior to and during transition to metabolic disease. Significant differences in plasma levels of total TAG, HDLs, HDL cholesterol, HDL large particles, HDL small particles, and IDL, shown as medians and IQR in metabolic impaired (Met^i^) and age- and weight-matched controls (Ctrl) at time of diagnosis (Dx) and 2 years prior to diagnosis (pre-Dx); n = 8 per group per time point. Variables shown are constituents of lipoprotein biomarker models (models 1 and 2). Statistical significance based on univariate analysis (two-sample Wilcoxon): * *P* < 0.05 for control versus impaired; Δ* *P* < 0.05 for difference between the changes for impaired and control animals from 2 years prior to time of diagnosis (Met^i^_Dx_-Met^i^_pre-Dx_) versus (Ctrl_Dx_-Ctrl_pre-Dx_).

To understand if progression to insulin resistance is also associated with differences in circulating lipoproteins, we conducted NMR-based lipoprotein profiling for healthy and (pre-)impaired animals. As part of the commercial package measures of total TAG, total cholesterol, HDL cholesterol, and CRP were also conducted. In humans, elevation of serum TAG has been shown to be tightly associated with metabolic syndrome (24, 25). Total TAGs were significantly elevated in impaired animals (OR, 31.37; *P* = 0.0006) at time of diagnosis (**Fig. 2**), and this in combination with insulin sensitivity data (Table 1) and biometric data (Fig. 1) satisfies established rhesus monkey criteria for metabolic syndrome (10): insulin resistance, plasma TAG >80 mg/dl, and adiposity in excess of 25%. At the time of diagnosis, total HDLs were significantly lower in plasma from impaired animals compared with controls (OR, 0.07; *P* = 0.002) (Fig. 2), LDL levels were unchanged, and VLDL and chylomicrons were significantly higher (OR, 6.77; *P* = 0.008), not shown. Total plasma cholesterol levels were not significantly different between groups at either time point (not shown); however, HDL cholesterol was lower (OR, 0.151; *P* = 0.006) at time of diagnosis (Fig. 2). Unexpectedly, significant differences in total HDL and HDL cholesterol were also detected at the earlier time point (*P* = 0.044 and 0.019, respectively) when all of the animals were insulin sensitive. Size distribution of the lipoproteins was determined at both time points (Fig. 2; supplementary Fig. 1). The reduction in HDL at the earlier time point was reflected in a significant reduction in large particles, while at time of diagnosis a significant difference was detected for large and small (Fig. 2) but not medium particles so that the median overall size of HDL particles was unchanged. Levels of the proinflammatory molecule CRP were not different between groups or between time points, perhaps due to the early stage of disease progression investigated in the current study (26). To determine whether lipoprotein profiles alone could distinguish insulin-resistant metabolic syndrome animals from healthy controls, we conducted lasso logistical regression of aggregate data. Lasso logistic regression analysis identified two new models from aggregate lipoprotein profiling data (**Table 2**). The first lipoprotein biomarker model (model 1) predicts insulin resistance in preimpaired animals with 88% accuracy 2 years prior to diagnosis when animals were still insulin sensitive and included total HDL and IDL lipoproteins (Fig. 2), in addition to large HDL particles and HDL cholesterol. The second lipoprotein biomarker model (model 2) identified insulin resistance with 100% accuracy at time of diagnosis and included HDL and IDL as before but now with the addition of small HDL particles and total TAG. These data indicate that lipoprotein profile shifts occur very early in the development of insulin resistance and suggest that lipoprotein biomarkers, in particular HDL, may be clinically informative outside of their usual context in evaluating risk for cardiovascular disease (27, 28).

**TABLE 2.** Biomarker models for insulin resistance

	OR*^a^*	SD	Accuracy
Model 1: lipoprotein profile prediagnosis			
Moiety			
HDL total	−31.1	8.154	88%
HDL large particles	−13.2	2.817	
HDL cholesterol	−23.9	5.189	
IDL	−7.5	30.393	
Model 2: lipoprotein profile at diagnosis			
Moiety			
HDL total	−88.1	11.12	100%
HDL small particles	−100	2.15	
IDL	918.6	29.652	
TAGs	7,116.8	80.06	
Model 3: DAG composition at diagnosis			
Fatty acid			
C16:1	−61.4	0.569	100%
C18:2(n-6)	169.4	5.274	
C18:3(n-3)	−9.7	0.181	
C20:1(n-7)	−35.8	0.19	
Model 4: lipid metabolism index at diagnosis			
Moiety			
PL C18:0	65.2	237.8	100%
PL C18:3(n-6)	21.4	2.5	
TAG C14:0	1.0	3.4	
HDL total	−43.3	4.4	
HDL large particles	−13.5	1.9	
LDL large particles	15.8	126.0	
TAGs	42.3	80.1	
Adiponectin HMW	−1.3	128.5	

a OR, OR for change in 1 SD.

### Plasma lipid profiles identify metabolic syndrome onset

To investigate the impact of disease progression on circulating lipids, fatty acid composition was determined in five lipid classes isolated from plasma. Lipid were extracted and separated into CEs, FFAs, DAGs, PLs, and TAG lipid classes. Individual fatty acid species within each class were identified from gas chromatography spectra. Percent composition of fatty acids within lipid class was calculated from peak areas. Fatty acids from 10 up to 24 carbon units were detected, and chain length and degree of saturation were resolved including MUFAs and PUFAs. Fatty acid concentration (g/ml plasma) was derived by adjustment of peak areas against those of spiked heptadecanoic acid standards for each class.

**Fig. 3.**
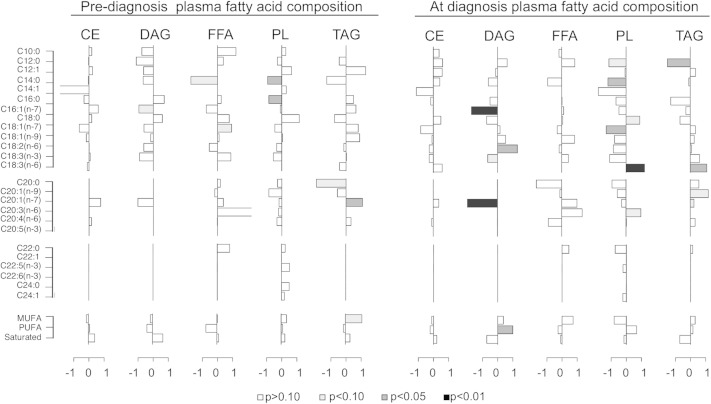
Plasma fatty acid composition profiles prior to and at onset of metabolic syndrome. Differences in fatty acid composition (percent of total species detected within class) of individual species between metabolic impaired and control groups prior to (left) and at time of diagnosis (right). Results are shown as the difference in medians divided by the median absolute deviation for each fatty acid species in plasma CE, DAG, FFA, PL, and TAG lipid classes. Species with bars that extend to the right are enriched in impaired animals compared with healthy animals; n = 8 per group per time point. Statistical significance based on univariate analysis (two-sample Wilcoxon) is shaded as indicated.

Two years prior to diagnosis, differences in fatty acid composition within lipid classes between healthy and preimpaired animals were modest; however, at time of diagnosis significant differences in plasma lipid composition were detected between healthy and insulin-resistant animals (**Fig. 3**). In the figure shown, difference in percent composition was calculated using medians [(pre-)impaired − healthy] and conducted for each time point. Data are shown as median differences adjusted to unit deviation where bars to the right are species increased in the impaired animals relative to healthy animals, and bars to the left are lower in impaired animals relative to healthy animals. For each individual fatty acid, the OR, confidence intervals, and *P* values were calculated using health status at time of diagnosis as the outcome. The impact of insulin resistance onset was specific to discrete fatty acid species within DAG, PL, and TAG lipid classes. Significant increases in levels of total, saturated, and unsaturated fatty acids were detected in these same classes at time of diagnosis (supplementary Fig. 2), and nonuniform increases in plasma levels of individual fatty acid species were detected within DAG, PL, and TAG in the impaired animals at time of diagnosis (supplementary Fig. 3). These data indicate that transition to insulin resistance impacts specific fatty acid species within specific lipid classes.

### DAG composition is the basis of a biomarker of insulin resistance

**Fig. 4.**
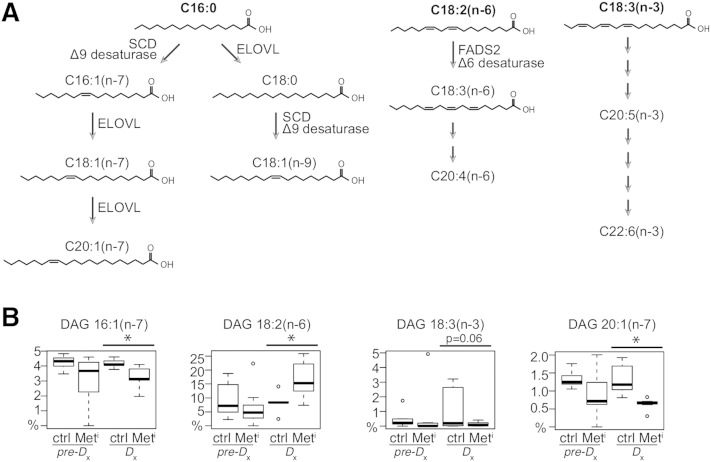
Changes in plasma DAG fatty acids with progression to metabolic syndrome. A: Schematic representation of fatty acid chain elongation and saturation from palmitic acid (C16:0) or dietary linoleic acid [C18:2(n-6)] and linolenic acid [C18:3(n-3)]. B: relative abundance of fatty acids that together constitute the DAG biomarker model. Data are shown as medians and IQR, and outliers are shown as open circles; n = 8 per group per time point. Statistical significance based on univariate analysis (two-sample Wilcoxon): * *P* < 0.05 for control versus impaired; Δ* *P* < 0.05 for difference between the changes for impaired and control animals from 2 years prior to time of diagnosis (Met^i^_Dx_-Met^i^_pre-Dx_) versus (Ctrl_Dx_-Ctrl_pre-Dx_).

Differences in fatty acid composition detected during progression to insulin resistance suggested that pathways of fatty acid elongation and desaturation may be differentially regulated in impaired animals (**Fig. 4A**). To determine whether fatty acid composition alone could distinguish insulin-resistant metabolic syndrome animals from healthy controls, we conducted lasso logistical regression of aggregate lipid-profiling data (all variables from all classes). A statistical model involving a single time point and a single lipid class that 100% correctly identifies metabolic impairment could have clinical value as an early biomarker. One such model (model 3) (Table 2) was identified from lipid-profiling data and was based on composition of DAG at time of diagnosis (Fig. 4B). Terms in the model included decreased relative concentrations of C16:1(n-7), the product of desaturation of palmitate; C18:2(n-6) and C18:3(n-3), essential fatty acids that were increased and decreased respectively; and C20:1(n-7), the elongation product of C18:1(n-7). Other statistical models with 100% accuracy in identifying insulin resistance at time of diagnosis included models based on the difference in the trajectory of change of healthy and impaired during disease progression requiring data from two time points, and thereby limiting their potential application as biomarkers of metabolic disease.

### Metabolic syndrome is associated with extensive changes in lipid metabolism

**Fig. 5.**
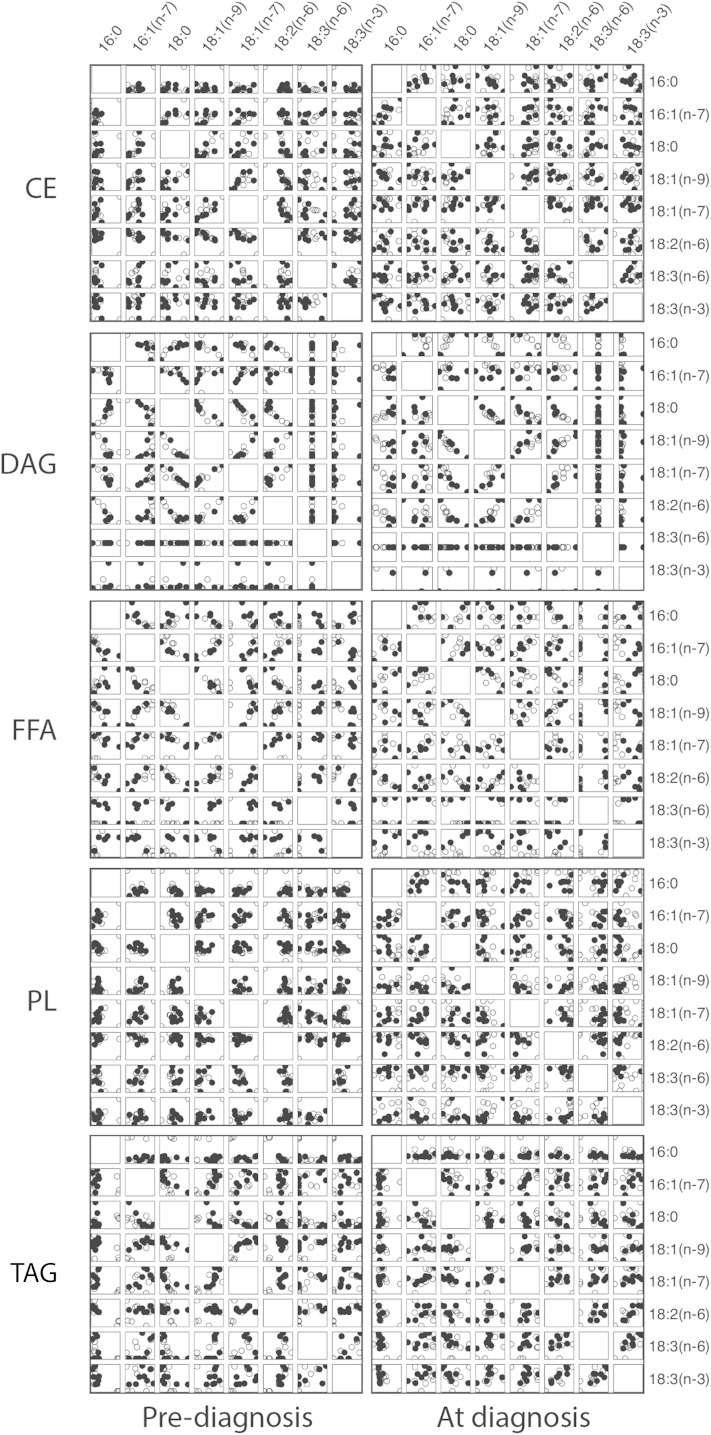
Lipid class-specific associations for fatty acid composition 2 years prior to and at time of diagnosis. Data are from healthy and impaired animals at time of diagnosis (right panels) and the same animals 2 years prior to diagnosis (left panels) when all animals were clinically identified as healthy. Fatty acid composition (percent of total species detected within class) data are plotted against each other for species from C16 and C18 chain length within lipid class. Lipid classes included plasma CE, DAG, FFA, PL, and TAG. Data are shown for control (open circles) and (pre-)impaired animals (filled circles); n = 8 per group per time point. Scales are not equivalent.

**Fig. 6.**
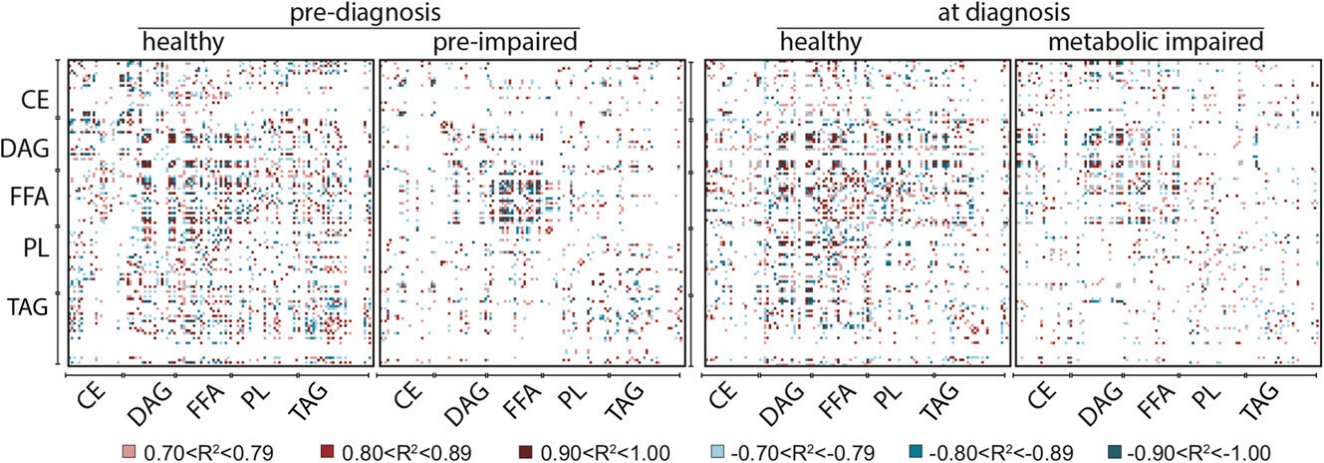
Correlations among plasma fatty acids reveal the lipid signature of metabolic syndrome. Large correlations of fatty acid composition (percent of total species detected within class) for fatty acids from plasma CE, DAG, FFA, PL, and TAG lipid classes calculated separately for healthy and impaired animals at time of diagnosis and 2 years prior when all animals were clinically identified as healthy. Positive and negative correlations are color coded as indicated; n = 8 per group per time point. The order of fatty acid species presented within each class from top to bottom and from left to right is as follows: C10:0, C12:0, C12:1, C14:0, C14:1, C16:0, C16:1, C18:0, C18:1(n-9), C18:1(n-7), C18:2(n-6), C18:3(n-6), C18:3(n-3), C20:0, C20:1(n-9), C20:1(n-7), C20:3(n-6), C20:4(n-6), C22:0, C22:5(n-3), C24:0, C24:1, saturated, MUFA, PUFA, where C24 species are detected in PL and TAG only. White areas indicate a correlation with absolute value ≤0.7 or species that fell below the threshold of detection.

To understand the relationships among fatty acid species, we calculated Spearman rank correlations between fatty acid species in terms of percent composition. Percent composition was plotted for C16 to C18 fatty acids against each other for each lipid class using pooled data from healthy and preimpaired animals at each time point, that is, 2 years prior to diagnosis when all animals were insulin sensitive (left) and the same animals at time of diagnosis when the impaired animals were insulin resistant (right) (**Fig. 5**). Strong positive and negative (absolute *R*^2^ ≥ 0.7) correlations were detected that were specific to fatty acids within lipid class and not equivalent among classes. For example, at the early time point (left panels) C18:2(n-6) and C18:1(n-9) were negatively correlated in CE, positively correlated in DAG, but not strongly correlated in FFA, PL, or TAG (Fig. 5; supplementary Table 2). Some correlations were detected at the early time point but not at time of diagnosis, for example C18:2(n-6) and C16:1(n-7) in FFA that were positively correlated at the early time point only (Fig. 5; supplementary Tables 2 and 3). We next calculated differences between correlations between healthy (open circles) and (pre-)impaired (filled circles) animals at both time points (Fig. 5; supplementary Table 4) to identify divergence in the associations among specific species of fatty acids at both time points. For example, in FFA at diagnosis a large difference in correlation values between healthy and impaired animals was detected for C18:2(n-6) and C18:1(n-9), where these species were strongly positively correlated in the impaired animals but were weakly negatively correlated healthy animals, while C18:2(n-6) and C18:0 were strongly negatively correlated in impaired animals and not correlated at all in healthy animals. To generate a means of visualizing the lipid signature of healthy and impaired animals at each time point, we calculated Spearman rank correlations across all fatty acids in all lipid classes and color coded the positive and negative correlation values as shown (**Fig. 6**). In healthy animals at both time points, strong correlations were detected broadly among lipid classes suggesting a high degree of connectivity in fatty acid composition. In contrast, in preimpaired animals strong correlations were clustered among fatty acids within FFA, at time of diagnosis strong correlations were detected among DAG and FFA, and at both time points fewer strong correlations among other lipid classes were detected in (pre-)impaired animals compared with healthy controls. These data suggest that differences in lipid metabolism in metabolic syndrome animals extend beyond the established clinical diagnostic of an increase in circulating levels of TAG and that changes may be occurring earlier than previously appreciated.

### Lipid metabolic index identifies insulin-resistant animals

**Fig. 7.**
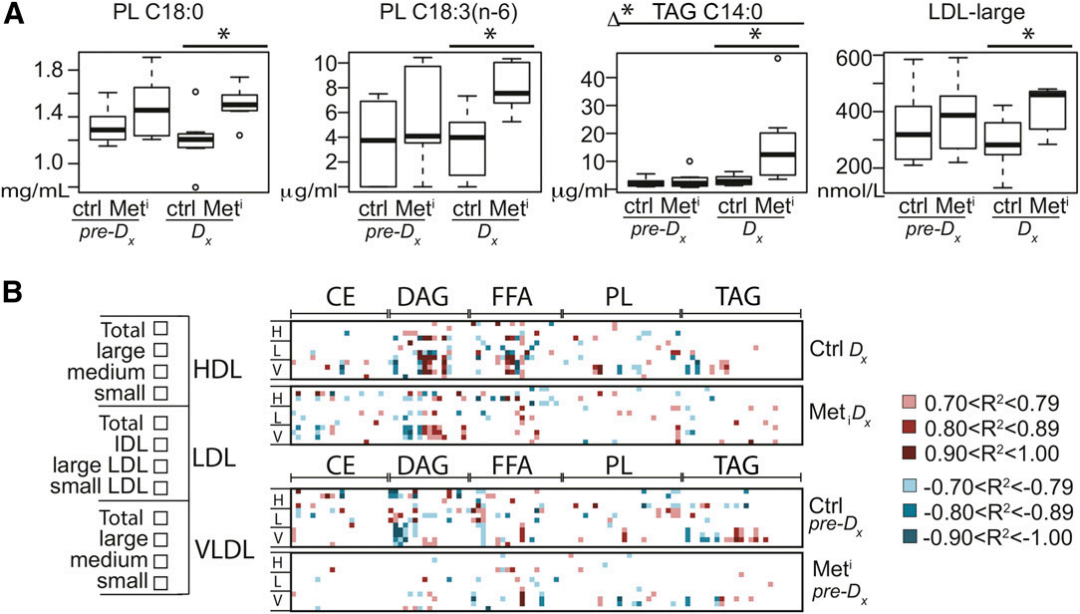
Lipid metabolic index connects fatty acid composition, lipoproteins, and adipokines to insulin resistance. A: Plasma levels of PL C18:0, PL C18:3(n-6), TAG C14:0, and LDL large particles shown as medians and IQR in metabolic impaired (Met^i^) and age- and weight-matched controls (Ctrl) at time of diagnosis (Dx) and 2 years prior to diagnosis (pre-Dx). Statistical significance based on univariate analysis (two-sample Wilcoxon) * *P* < 0.05 control versus impaired; Δ* *P* < 0.05 for difference between the changes for impaired and control animals from 2 years prior to time of diagnosis (Met^i^_Dx_-Met^i^_pre-Dx_) versus (Ctrl_Dx_-Ctrl_pre-Dx_). Variables shown are constituents of lipid index biomarker model. B: Large correlations of lipoprotein size distribution (listed left) with fatty acid percent composition calculated separately for healthy and impaired animals at both time points. Positive and negative correlation values are color coded as indicated; n = 8 per group per time point. Lipid classes include CE, DAG, FFA, PL, and TAG. The order of fatty acid species (from left to right within each class) follows: C10:0, C12:0, C12:1, C14:0, C14:1, C16:0, C16:1, C18:0, C18:1(n-9), C18:1(n-7), C18:2(n-6), C18:3(n-6), C18:3(n-3), C20:0, C20:1(n-9), C20:1(n-7), C20:3(n-6), C20:4(n-6), C22:0, C22:5(n-3), C24:0, C24:1, saturated, MUFA, PUFA, where C24 species are detected in PL and TAG only. White areas indicate a correlation with absolute value ≤0.7 or species that fell below the threshold of detection.

Next, we conducted lasso logistic regression analysis across the entire data set including body composition, adipokine levels, fatty acid levels and percent composition, and lipoprotein profiles to identify potential predictors. Together the variables featured in biomarker model 4 (Table 2) identified insulin resistance at time of diagnosis with 100% accuracy. Terms in the model include individual fatty acid species from PL and TAG (**Fig. 7A**) in addition to HDL, LDL, and HMW adiponectin. All terms with the exception of HMW adiponectin were independently significantly different between healthy and impaired animals. Terms that are not themselves significantly different contribute to the joint model when they are correlated with other terms that are different between conditions. To investigate whether differences in lipoprotein profiles were linked with differences in fatty acid percent composition, we calculated strong correlations (*R*^2^ ≥ 0.7 absolute value) for HDL, LDL, and VLDL, including particle size distribution, and fatty acid percent composition. To visualize the lipoprotein-lipid signature, correlations were color coded as before (Fig. 7B). At time of diagnosis (upper two panels), strong correlations for lipoproteins from LDL, VLDL, and small HDL were clustered among DAG and FFA for healthy animals. Fewer strong correlations were detected in the impaired animals. Two years prior to diagnosis (lower two panels), strong correlations were clustered within the DAG for healthy animals, and again, fewer strong correlations were observed in the preimpaired animals across all lipid classes. These data suggest that the associations between lipoprotein profiles and fatty acid composition are not static over time and are responsive to differences in insulin sensitivity.

## DISCUSSION

In the course of this study, it became apparent that the insulin-resistant animals fit the criteria for metabolic syndrome; however, the overarching goal of the study was to identify lipid-related events in the early stages of spontaneous insulin resistance development. Animals were monitored for glucoregulatory function every 6 months, equivalent to ∼1.5 human years, ensuring that the data generated from the diagnosed animals capture the metabolic context early in disease development. Diagnosis was based on fasting insulin >70 μU/ml and Si <2(E−04). In clinical settings, glucoregulatory impairment is often first identified by abnormally elevated fasting blood glucose levels (100–125 mg/dl) and/or abnormally elevated fasting blood glycosylated hemoglobin (HbA1c > 5.7–6.4%), a condition known as IFG or prediabetes. Fasting plasma glucose was not different between groups at either time point confirming that, similar to humans (29, 30), loss of insulin sensitivity occurs early in disease progression in rhesus monkeys in advance of hyperglycemia. The increased risk for metabolic impairment conferred by elevated adiposity is long established (31, 32), but the possibility that the quality of adipose tissue is more important in disease vulnerability than the extent of adiposity informed the decision to use pair-matched overweight metabolically impaired and control animals. In the course of the study, we showed that adiposity, abdominal circumference, and percent abdominal adiposity were not significantly different between healthy and impaired animals at time of diagnosis or 2 years earlier. This suggests that the biomarkers presented herein, if conserved between humans and nonhuman primates, will be effective in identifying insulin resistance among overweight individuals in advance of IFG.

The biomarker models of insulin resistance identified in this study pertain to lipid metabolism and lipid transport. The DAG biomarker model is based on changes in relative composition of fatty acid species within the class. A change in DAG composition suggests that pathways of fatty acid elongation and desaturation are differentially regulated during disease progression; however, critical tissues precipitating differences in plasma DAG composition are currently unknown. Data presented here suggest that DAG composition may reflect differences in underlying lipid metabolism between healthy and impaired animals. The identification of DAG composition as a biomarker does not inform us of any potential causal role in disease etiology; any signaling capability or bioactivity of plasma DAG has yet to be described. The lipoprotein biomarker models are consistent with what is known about metabolic disease in human health but suggest that lipoprotein profiling may be informative for risk assessment early in disease development. In human studies, an imbalance between circulating levels of HDL relative to LDL and VLDL is associated with systemic inflammation (33). A decline in HDL levels has previously been associated with elevated risk for cardiovascular disease, and lipoprotein profiling has also been used in evaluating risk for atherosclerosis (28). The current study points to the potential importance of lipoprotein size distribution early in insulin resistance and metabolic syndrome development. Changes in HDL levels and size distribution early in the development of insulin resistance have not been previously shown, but smaller and denser subclasses of HDL are thought to have greater antioxidant activity and superior anti-inflammatory properties than the larger classes (34, 35). The multiterm biomarker model (model 4) encompasses variables generated from lipid, lipoprotein, and adipokine profiling. In terms of clinical application, it is less likely to be of value considering the burden of analysis required. In terms of biology, the multiterm model indicates that each of the components connect in some way to the development of insulin resistance and strongly suggests that changes in lipid metabolism contribute to spontaneous insulin resistance development.

Adipose tissue contributes to circulating lipid composition and responds to differences in circulating lipid composition (36, 37). There is a reasonable possibility that adipose tissue function contributes to disease vulnerability even in the absence of differences in adiposity. Adiponectin was one of the terms identified in the lipid index biomarker model, and recent literature supports the concept that this adipose-derived molecule could contribute to differences in plasma lipid composition associated with insulin resistance (38–41). In seeking to identify the nature of the changes in lipid metabolism, we conducted a series of regression analyses. In the first analysis limited to percent composition of C16-C18 species within lipid classes, animals were grouped by time point so that divergence over time could be evaluated longitudinally. These data revealed a high degree of lipid class specificity in the associations among fatty acids. Relationships among fatty acid species within lipid class are likely to be influenced by the tissue source of the lipids and the substrate preferences of resident enzymes involved in elongation, desaturation, and multicomponent lipid assembly. Changes in correlations during development of insulin resistance were also lipid class specific, indicating that disease development influences multiple regulatory steps in assembly of multicomponent lipids. In addition to lipid class specificity in the relationships among fatty acids species, a complex relationship among lipid classes was revealed in the extended regression analysis. The identification of multiple correlations across classes suggests that there is a system-wide coordination of lipid processing and synthesis in healthy animals. This contrasted with the lipid signature of (pre-)impaired animals where correlations were clustered within FFA or DAG, suggesting that the higher level of order is lost in impaired animals. Possible contributing processes occurring in one or more tissues include changes in substrate preference for multicomponent lipid assembly, differences in the composition of the fatty acid pool, or changes in turnover of select species, any of which alone or in combination would be predicted to impact system-level coordination. In the lipoprotein-lipid signature of healthy animals, correlations between DAG and FFA and lipoprotein profiles were prominent; however, fewer correlations were detected for the (pre-)impaired animals in both classes at both time points compared with healthy controls. These data demonstrate that there are phase transitions in the lipid and lipoprotein-lipid signatures of disease progression and suggest that DAG composition may reflect complex differences in the underlying lipid biology.

Lipid profiling has been used extensively in the context of cardiovascular disease (42) and more recently metabolic syndrome (43). Differences in plasma fatty acid composition have been reported in human studies focusing on a later stage in metabolic syndrome progression (44–47). Notwithstanding the early stage in disease progression that is the focus of the current study, many of the changes previously identified in humans are recapitulated in this rhesus monkey study. The reduction in HDL levels and the increases in levels of DAG, PL, and TAG reported here have also been detected in metabolic syndrome in humans (45). The data presented here suggest that changes in lipid metabolism and lipid transport occur early in disease progression, certainly by the time insulin resistance has developed, and possibly even in advance of that. Given the high degree of conservation between humans and rhesus monkeys, we propose that these findings are likely to be highly translatable to human metabolic disease and suggest that a lipid-based biomarker may be informative as a clinical assessment of metabolic syndrome risk in overweight individuals.****

## Supplementary Material

Supplemental Data
