# Supplementary material for: Plasma diacylglycerol composition is a biomarker of metabolic syndrome onset in rhesus monkeys
Source: J Lipid Res. 2015 Aug;56(8):1461–70. doi: 10.1194/jlr.M057562 (PMC4513987; doi:10.1194/jlr.M057562)
Supplement: Supplemental Data [file supp_56_8_1461__index.html]

Plasma diacylglycerol composition is a biomarker of metabolic syndrome onset in rhesus monkeys — Plasma diacylglycerol composition is a biomarker of metabolic syndrome onset in rhesus monkeys — Supplemental Data 

# Plasma diacylglycerol composition is a biomarker of metabolic syndrome onset in rhesus monkeys

## Supplemental Data

- Supplemental material combined - Supplementary material combined
