# Supplementary material for: Plasma diacylglycerol composition is a biomarker of metabolic syndrome onset in rhesus monkeys
Source: J Lipid Res. 2015 Aug;56(8):1461–70. doi: 10.1194/jlr.M057562 (PMC4513987; doi:10.1194/jlr.M057562)
Supplement: Supplemental Data [file supp_M057562_jlr.M057562-1.pdf]

Supplementary information

Figure Legends

Figure S1. Lipoprotein profiles are altered prior to and during transition to metabolic disease. Significant differences in plasma levels of LDL (low density lipoproteins), VLDL (very low density lipoproteins), and lipoprotein size shown as medians and IQR in metabolic impaired (Meti) and age and weight matched controls (Ctrl) at time of diagnosis (Dx) and two years prior to diagnosis (pre-Dx), n=8 per group per time point. Statistical significance based on univariate analysis (two sample Wilcoxon) * $p<0.05$ for control vs impaired, Δ* p value <0.05 for difference between the changes for impaired and control animals from two years prior to time of diagnosis ($Meti_{Dx}$-$Meti_{preDx}$) vs ($Ctrl_{Dx}$-$Ctrl_{preDx}$).

Figure S2. Plasma fatty acid levels and degree of saturation are altered with onset of metabolic syndrome. Concentrations of indicated fatty acid (FA) species in plasma cholesterol ester (CE), diacyglycerol (DAG), free fatty acid (FFA), phospholipid (PL), and triacylglycerol (TG) lipid classes. Data are shown as medians and IQR in metabolic impaired (Meti) and age and weight matched controls (Ctrl) at time of diagnosis (Dx) and two years prior to diagnosis (pre-Dx). CE:n=8,8 (healthy, impaired respectively); DAG:n=5,7; FFA: n=5,7; PL:n=8,8; TG:n=8,8 at each time point. Statistical significance based on univariate analysis (two sample Wilcoxon) * $p<0.05$ for control vs impaired, Δ* p value <0.05 for difference between the changes for impaired and control animals from two years prior to time of diagnosis ($Meti_{Dx}$-$Meti_{preDx}$) vs ($Ctrl_{Dx}$-$Ctrl_{preDx}$).

Figure S3. Lipid class specific changes in plasma fatty acid concentrations with onset of metabolic syndrome. Differences in fatty acid concentration of individual species between metabolic impaired and control groups prior to (left) and at time of diagnosis (right). Results are shown as the difference in medians divided by the median absolute deviation for each fatty acid species in plasma cholesterol ester (CE), diacyglycerol (DAG), free fatty acid (FFA), phospholipid (PL), and triacylglycerol (TG) lipid classes. Species with bars that extend to the right are enriched in impaired animals compared to healthy animals, CE:n=8,8 (healthy, impaired respectively); DAG:n=5,7; FFA: n=5,7; PL:n=8,8;

TG:n=8,8 at each time point. Statistical significance based on univariate analysis (two sample Wilcoxon) is shaded as indicated.

Supplementary Table 1 Study Cohort (medians and quartiles)

At time of diagnosis

	Age (y)	Wt (Kg)	Glucose	Insulin	Si (E-04)*
**Healthy**	14.96 (13.87, 20.12)	14.24 (12.40, 15.69)	63.0 (58.5, 66.5)	**33.0 (24.0, 54.5)**	**2.26 (1.5, 5.37)**
**Impaired**	16.39 (14.88, 21.89)	15.51 (13.34, 16.80)	66.5 (61.5, 90)	**133.5 (88.75, 169.75)**	**0.12 (0, 0.54)**

Two years prior to diagnosis

	Age (y)	Wt (Kg)	Glucose	Insulin	Si (E-04)*
**Healthy**	12.96 (11.86, 18.15)	13.09 (12.25, 14.62)	61.0 (55.5, 64)	29.0 (20.5, 48.5)	1.63 (1.3, 5.29)
**Pre-Impaired**	14.40 (12.87, 19.89)	13.20 (12.88, 15.84)	62.0 (60.75, 66.5)	45.5 (37.25, 58.75)	1.6 (1.43, 2.1)

Biometric data median (IQR) (n=8 per group), values in bold are significantly different $p<0.05$
*Insulin sensitivity (Si (E-04)) was generated by the modified minimal model approach utilizing data from the intravenous frequently sampled glucose tolerance test.

Supplementary Table 2 Correlations among fatty acids by composition in lipid class where absolute correlations is ≥ 0.7

	Pre-diagnosis (n=16)								At diagnosis (n=16)							
**Fatty acid**	**16:0**	**16:1(n-7)**	**18:0**	**18:1(n-9)**	**18:1(n-7)**	**18:2(n-6)**	**18:3(n-6)**	**18:3(n-3)**	**16:0**	**16:1(n-7)**	**18:0**	**18:1(n-9)**	**18:1(n-7)**	**18:2(n-6)**	**18:3(n-6)**	**18:3(n-3)**
CE.16:0																
CE.16:1(n-7)			0.8			-0.75										
CE.18:0		0.8				-0.84								-0.74		
CE.18:1(n-9)					0.72	-0.89										
CE.18:1(n-7)				0.72		-0.75										
CE.18:2(n-6)		-0.75	-0.84	-0.89	-0.75						-0.74					
CE.18:3(n-6)																
CE.18:3(n-3)																
DAG.16:0			0.83								0.76	-0.74		-0.8		
DAG.16:1																
DAG.18:0	0.83			-0.87	-0.93	-0.83			0.76			-0.88	-0.83	-0.94		
DAG.18:1(n-9)			-0.87		0.85	0.77			-0.74		-0.88		0.73	0.85		
DAG.18:1(n-7)			-0.93	0.85		0.78					-0.83	0.73		0.74		
DAG.18:2(n-6)			-0.83	0.77	0.78				-0.8		-0.94	0.85	0.74			
DAG.18:3(n-6)																
DAG.18:3(n-3)																
FA.16:0		-0.88		-0.85		-0.71		0.71						-0.81		
FA.16:1(n-7)	-0.88		-0.82	0.88		0.82							0.87			
FA.18:0		-0.82				-0.87						-0.84				
FA.18:1(n-9)	-0.85	0.88						-0.75			-0.84					
FA.18:1(n-7)										0.87						
FA.18:2(n-6)	-0.71	0.82	-0.87						-0.81							
FA.18:3(n-6)																
FA.18:3(n-3)	0.71			-0.75												
PL.16:0																
PL.16:1(n-7)																
PL.18:0																
PL.18:1(n-9)																
PL.18:1(n-7)																
PL.18:2(n-6)																
PL.18:3(n-6)																
PL.18:3(n-3)																
TG.16:0						-0.79									-0.72	
TG.16:1(n-7)													0.75			
TG.18:0				-0.74	-0.76		-0.8									
TG.18:1(n-9)			-0.74													
TG.18:1(n-7)			-0.76							0.75						
TG.18:2(n-6)	-0.79							0.85								
TG.18:3(n-6)			-0.8						-0.72							
TG.18:3(n-3)						0.85										

Supplementary Table 3 Difference in correlations for fatty acid composition between time points by lipid class where the absolute differences is ≥ 0.5

At diagnosis-pre diagnosis (n=16)								
**Fatty acid**	**16:0**	**16:1(n-7)**	**18:0**	**18:1(n-9)**	**18:1(n-7)**	**18:2(n-6)**	**18:3(n-6)**	**18:3(n-3)**
CE.16:0								
CE.16:1(n-7)				-0.83				
CE.18:0								
CE.18:1(n-9)		-0.83						
CE.18:1(n-7)								
CE.18:2(n-6)								
CE.18:3(n-6)								
CE.18:3(n-3)								
DAG.16:0		0.71						
DAG.16:1	0.71		0.6					
DAG.18:0		0.6						
DAG.18:1(n-9)								0.57
DAG.18:1(n-7)								0.76
DAG.18:2(n-6)								
DAG.18:3(n-6)								
DAG.18:3(n-3)				0.57	0.76			
FA.16:0		0.93						
FA.16:1(n-7)	0.93		0.64	-0.69	0.56	-1.03		
FA.18:0		0.64						
FA.18:1(n-9)		-0.69						
FA.18:1(n-7)		0.56						
FA.18:2(n-6)		-1.03						
FA.18:3(n-6)								
FA.18:3(n-3)								
PL.16:0		0.59						
PL.16:1(n-7)	0.59			-0.77				-0.53
PL.18:0				-0.72			0.56	-0.75
PL.18:1(n-9)		-0.77	-0.72		-0.67	0.65		
PL.18:1(n-7)				-0.67				
PL.18:2(n-6)				0.65				0.64
PL.18:3(n-6)			0.56					
PL.18:3(n-3)		-0.53	-0.75			0.64		
TG.16:0			-0.56					
TG.16:1(n-7)								
TG.18:0	-0.56			0.52				
TG.18:1(n-9)			0.52					
TG.18:1(n-7)						-0.59		
TG.18:2(n-6)					-0.59			
TG.18:3(n-6)								
TG.18:3(n-3)								

Supplementary Table 4 Difference in correlations for fatty acid composition between healthy and impaired by lipid class by time point where the difference is ≥ 0.5

	Pre-diagnosis (n=16)								At diagnosis (n=16)							
**Fatty acid**	**16:0**	**16:1(n-7)**	**18:0**	**18:1(n-9)**	**18:1(n-7)**	**18:2(n-6)**	**18:3(n-6)**	**18:3(n-3)**	**16:0**	**16:1(n-7)**	**18:0**	**18:1(n-9)**	**18:1(n-7)**	**18:2(n-6)**	**18:3(n-6)**	**18:3(n-3)**
CE.16:0		0.67	0.76				0.77					-1.19	0.5	0.52	0.79	
CE.16:1(n-7)	0.67							0.67				-0.76			0.6	
CE.18:0	0.76							0.76				-0.69		0.6		
CE.18:1(n-9)									-1.19	-0.76	-0.69		-0.62			
CE.18:1(n-7)									0.5			-0.62				-0.62
CE.18:2(n-6)									0.52		0.6					
CE.18:3(n-6)	0.77							0.77	0.79	0.6						
CE.18:3(n-3)													-0.62			
DAG.16:0											0.83	-0.69	-0.69	-0.66		
DAG.16:1					0.58									-0.63		
DAG.18:0									0.83							
DAG.18:1(n-9)								-0.67	-0.69							-0.67
DAG.18:1(n-7)		0.58							-0.69							
DAG.18:2(n-6)									-0.66	-0.63						
DAG.18:3(n-6)																
DAG.18:3(n-3)				-0.67								-0.67				
FA.16:0					-0.7							-0.62				
FA.16:1(n-7)					0.99							-0.95			-1.08	
FA.18:0				-0.56										-0.93		
FA.18:1(n-9)			-0.56		1.35				-0.62	-0.95			-0.76	1.56		
FA.18:1(n-7)	-0.7	0.99		1.35		0.67	0.62	-0.95				-0.76		0.99	-1.02	
FA.18:2(n-6)					0.67						-0.93	1.56	0.99		0.57	-0.64
FA.18:3(n-6)					0.62					-1.08			-1.02	0.57		
FA.18:3(n-3)					-0.95									-0.64		
PL.16:0		0.6		0.62						-0.52	-0.64			1.05	0.66	0.52
PL.16:1(n-7)	0.6		-0.74						-0.52			-0.79	0.55			
PL.18:0		-0.74		-0.79				-1.12	-0.64							
PL.18:1(n-9)	0.62		-0.79							-0.79				0.86		
PL.18:1(n-7)								-0.57		0.55					0.79	
PL.18:2(n-6)							-0.55	0.64	1.05			0.86				0.71
PL.18:3(n-6)						-0.55			0.66				0.79			
PL.18:3(n-3)			-1.12		-0.57	0.64			0.52					0.71		
TG.16:0		0.55		0.83	1.07						0.86		0.52			
TG.16:1(n-7)	0.55										-0.57			0.83		
TG.18:0									0.86	-0.57		-0.5	-0.55	-0.86	-1.26	-1.19
TG.18:1(n-9)	0.83										-0.5		-0.79			
TG.18:1(n-7)	1.07					-1.1	-0.6		0.52		-0.55	-0.79				
TG.18:2(n-6)					-1.1			-0.5		0.83	-0.86					
TG.18:3(n-6)					-0.6						-1.26					
TG.18:3(n-3)						-0.5										

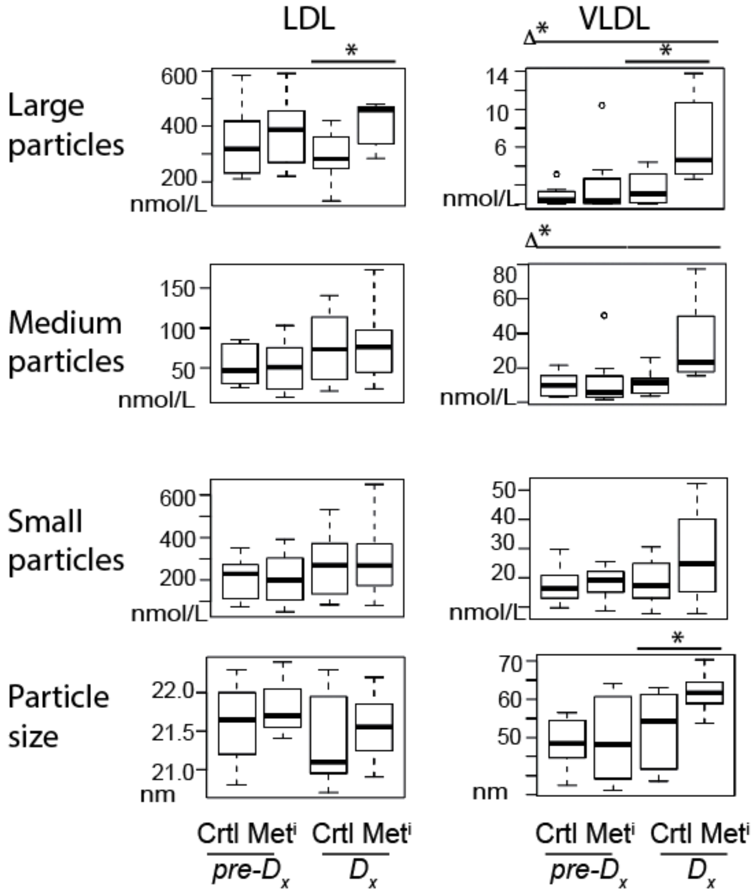

Supplementary Figure 1

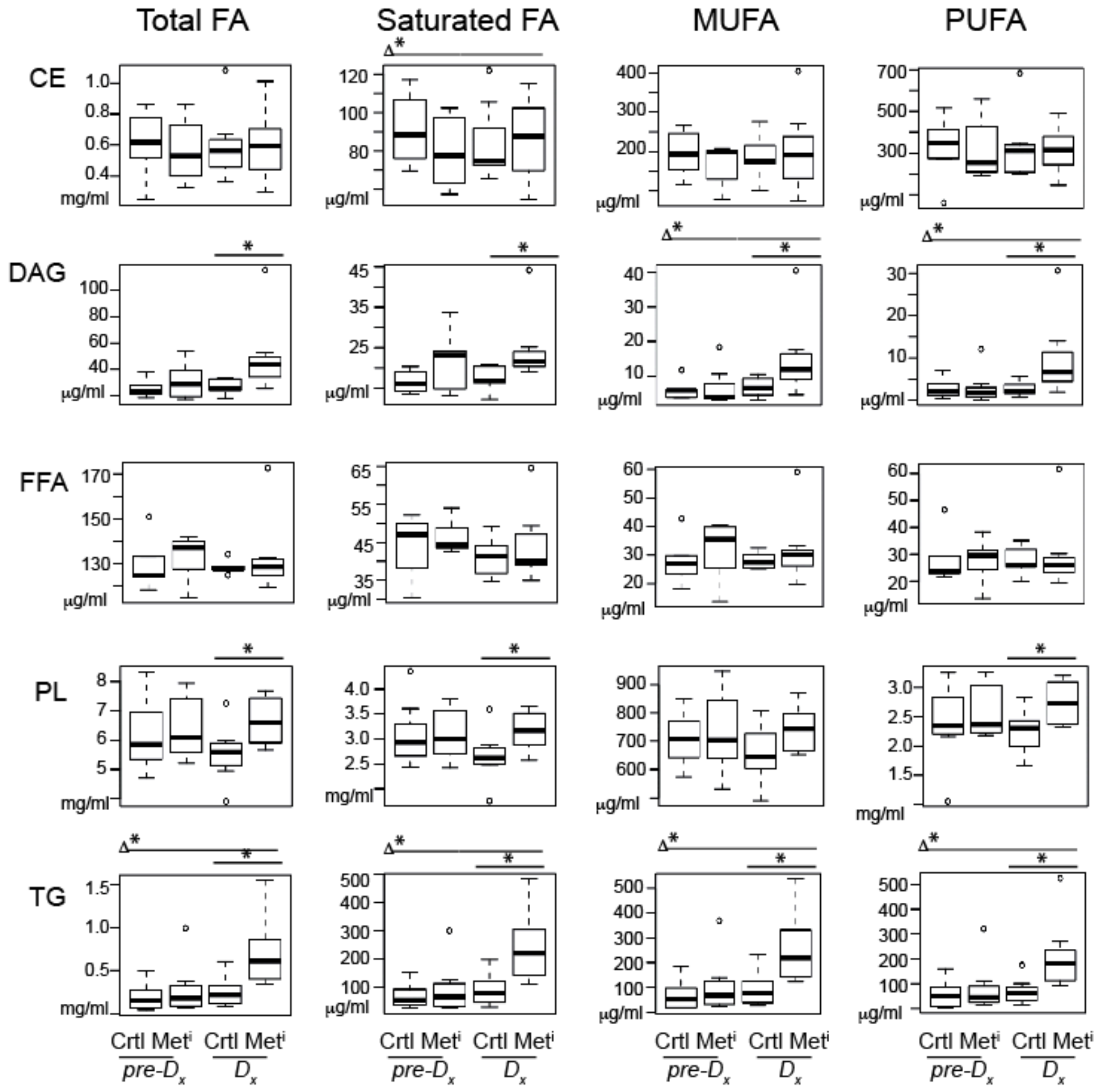

Supplementary Figure 2

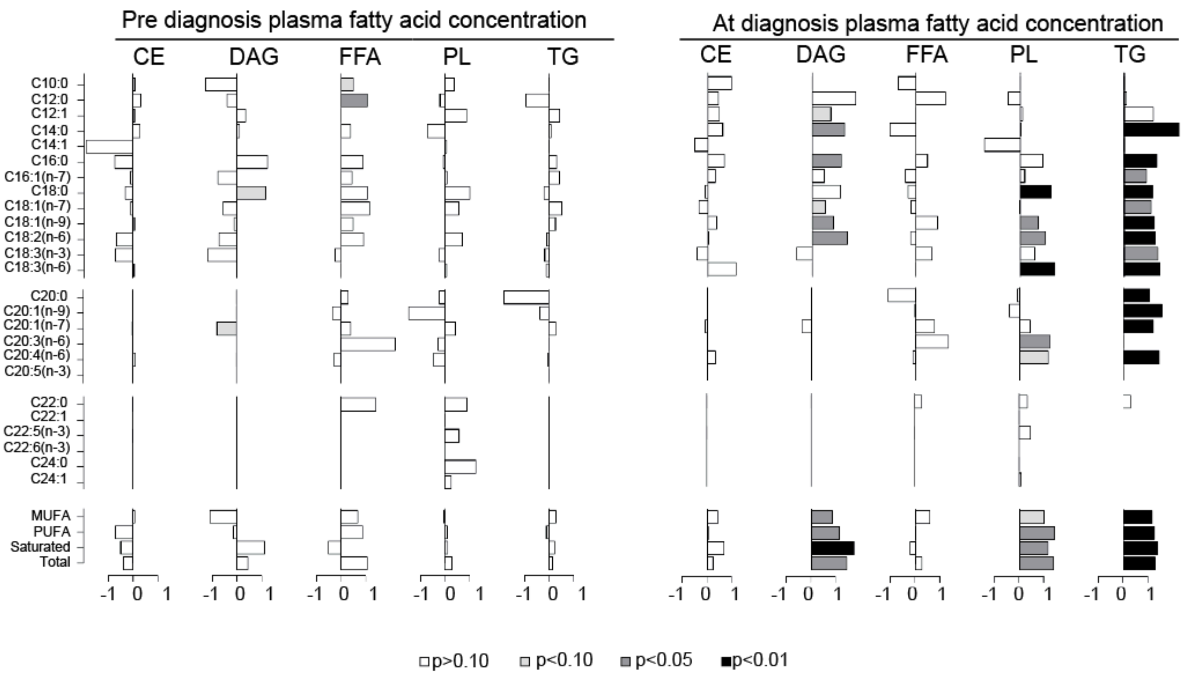

Supplementary Figure 3
